# Size-based niche partitioning permits coexistence in natural populations of *Nicrophorus* spp.

**DOI:** 10.1093/ee/nvaf087

**Published:** 2025-10-28

**Authors:** Andrew Martin Catherall-Ostler

**Affiliations:** Homerton College, University of Cambridge, Cambridge, UK

**Keywords:** interspecific competition, niche partitioning, sexual dimorphism

## Abstract

When species compete over similar resources, niche partitioning can permit ecologically similar species to coexist. Such coexistence should be a particular challenge for carrion-feeding invertebrates, with the ephemeral nature of carrion leading to intense competition over this nutrient-rich resource. Here we tested whether the carrion niche in four species of coexisting burying beetles (*Nicrophorus* spp.) is partitioned seasonally or by species size in seven ancient woodlands in the United Kingdom. We fail to replicate the results of previous studies in the UK that found a strict seasonal separation in the activity of competing burying beetle species. Instead, our data support the hypothesis that the niche is partitioned by species size. We present field evidence consistent with the hypothesis that smaller species are less successful at contested carcasses and confirm that sexual dimorphism in head width, a trait likely related to competitive ability, is present in several species of *Nicrophorus*. We discuss the considerable but as-of-yet unnoticed variation between different geographic populations of *Nicrophorus* spp. in how the carrion niche is partitioned.

## Introduction

Understanding how ecologically similar species can coexist has long been a key challenge for community ecologists (Hutchinson 1957, Macarthur 1965, Chesson 2000). When species compete over the same resources, either the competitively stronger species will eliminate weaker ones (Pimentel et al. 1965, MacArthur and Wilson 1967, Bengtsson 1989) or the niche will be partitioned, allowing coexistence (Gause 1932, Hutchinson 1957, Hardin 1960). Theory predicts that the balance of intraspecific and interspecific competition will determine whether or not coexistence occurs: stable coexistence is only possible when intraspecific competition is more intense than intraspecific competition (Vandermeer and Goldberg 2013, Barabás et al. 2016, Mittelbach and McGill 2019).

The communities that feed on carrion are an excellent system in which to investigate species coexistence. Carrion exists in all ecosystems (Barton et al. 2013, Benbow et al. 2015), providing a nutrient-rich resource that is consumed by up to 10% of vertebrates (Mateo‐Tomás et al. 2015) and an even more diverse guild of invertebrate species (Anderson et al. 2019). High levels of invertebrate diversity at carrion is the norm rather than the exception (Olea et al. 2019): for example, Barry et al. (2019) recorded 215 beetle species on 18 ungulates killed by pumas in Yellowstone, United States. The diversity of invertebrate carrion-feeding communities is surprising for two reasons. Firstly, as all are feeding on the same resource, the level of niche overlap is predicted to be high (Olea et al. 2022). Secondly, the ephemeral nature of carrion in time and space means that competition for this high-quality bonanza resource is often intense (Wilson 1971, Tallamy and Wood 1986, Trumbo 2016).

Burying beetles (*Nicrophorus* spp.) provide a good system in which to investigate how the carrion niche may be partitioned. Fast becoming a model organism in evolutionary biology (Jarrett 2018), the genus contains 73 described species (Sikes and ­Venables 2013, Sikes et al. 2016) distributed principally across northern temperate habitats (Sikes 2005) and is characterized by an elaborate repertoire of parental behaviors (Pukowski 1933, Scott 1998, Royle et al. 2013). For most species the life cycle centres around the discovery of small vertebrate carrion: adults use chemical cues to locate the carcasses of small mice or birds upon which they attempt to breed (Kalinová et al. 2009). As carrion is scarce, it is normal for many competing burying beetles to arrive in quick succession at a newly deceased corpse, with intrasexual fights taking place until the carcass is secured by a single dominant pair comprising the winners within each sex (Eggert and Müller 1997, Trumbo 2016). The carcass is then converted into an edible nest upon which the dominant pair will raise their young.

Coexistence in burying beetles could be permitted if the carrion niche is partitioned temporally, i.e. if species were active at different times of day or at different points across the year. Phenological patterns consistent with seasonal niche partitioning have been discovered in burying beetles, but this work has focused mainly on North American and Japanese species (Scott 1998, Keller et al. 2019). In the United Kingdom, 6 species are resident (*N. germanicus* (Linnaeus, 1758) (Coleoptera: Silphidae), *N. humator* (Gleditsch, 1767), *N. interruptus* (Stephens, 1830), *N. investigator* (Zetterstedt, 1824), *N. vespillo* (Linnaeus, 1758) and *N. vespilloides* (Herbst, 1783), and there seems to be considerable geographic variation in the way the niche is partitioned temporally. Springett (1967) found a near-total separation of activity periods between *N. humator* and *N. investigator* in Northumberland: in the weeks where *N. investigator* was active, *N. humator* was absent and vice-versa. In contrast, Easton (1979) found the two species overlapped in Stirlingshire; discrepancies also exist between the activity periods reported across Western Europe (Dekeirsschieter et al. 2011) compared to those in Great Britain (Esh and Oxbrough 2021). A lack of data hampers a fuller understanding of the seasonal partitioning of the carrion niche in burying beetles: very little is known, for example, of the phenology of the rare *N. interruptus* (O’Hanlon et al. 2020), and only one large-scale quantitative study of the spatial and temporal distribution of burying beetles has been conducted in western Europe (Esh and Oxbrough 2021).

Instead, the leading hypothesis for coexistence in burying beetles is that the carrion niche is partitioned by species size, with competitively dominant larger species monopolizing more valuable larger carrion (Scott 1998, Hopwood et al. 2016, Potticary et al. 2024). The dominance of larger species arises due to the fierce fighting over carcasses that is common in natural populations (Eggert and Müller 1997, Scott 1998, Trumbo 2016): fighting ability is positively associated with body size (Otronen 1988, Royle and Hopwood 2017).

Previous work in two British woodlands found that the four species resident do indeed differ in size from another (*N. vespilloides* < *N. interruptus* < *N. investigator* < *N. humator*; Sun (2020)), though these size differences have not yet been replicated. If the size differences are robust, then this implies coexistence is permitted through each occupying its own distinct size niche, with this in turn limiting the size of carrion it breeds on: competitively inferior smaller species would be limited to smaller carrion, whilst larger species can secure carcasses of any size through physical combat but prefer larger carcasses due to their greater reproductive potential. If multiple species arrive at intermediate-sized carrion on which all could breed (i.e. at a resource where there is niche overlap), then we predict that the larger species should prevail, but field evidence that larger *Nicrophorus* species actually do pose a competitive threat to smaller species is lacking.

It is also questionable whether pronotal width comparisons are the best proxy for differences in competitive ability. Whilst pronotal width is the standard measure of burying beetle body size (Bartlett and Ashworth 1988, Müller et al. 1990, Safryn and Scott 2000, Hopwood et al. 2013, 2014, Lee et al. 2014), actual observations of contest behavior suggest that the combatants primarily use their mandibles to fight—not their pronota. Warring beetles bite viciously at each other’s abdomens, antennae, and legs, and it is not uncommon for fights to result in injury or death (Bartlett 1987, Müller et al. 2003, Eggert et al. 2008, Sakaluk and Müller 2008). If head width is a better proxy of competitive ability than pronotal width, then we would expect sex differences in its allometric slope due to males experiencing more intense selection for fighting ability than females (Eggert and Müller 1997, Trumbo 2006). Whilst burying beetles were traditionally thought to be sexually monomorphic, Smith et al. (2024) recently discovered that head width in *N. vespilloides* is sexually dimorphic, scaling hyperallometrically with pronotal width in males but isometrically in females. It has not been tested whether this scaling relationship is present in other *Nicrophorus* species. As hyperallometric scaling is a hallmark of biological weapons, head width may be a more relevant morphological trait to measure in the context of competition than pronotal width.

To investigate how the carrion niche is partitioned seasonally and morphologically, we carried out three years of burying beetle trapping data in seven ancient woodlands in Bedfordshire, Cambridgeshire, and Huntingdonshire (United Kingdom). We used this data to ask three specific questions:

1. *Are seasonal patterns in species abundance consistent with seasonal niche partitioning?*
2. *Do coexisting beetle species occupy different sized niches, as measured by pronotal and/or head width?*
3. *Does the arrival of a larger species at a trap increase the probability of smaller species dying?*

## Methods

### Trapping Programme

Trapping took place in seven ancient woods: Buff Wood (Cambridgeshire), Cockayne Hatley Wood (Bedfordshire), Gamlingay Wood (Cambs.), Hayley Wood (Cambs.), Waresley Wood (Huntingdonshire), and Weaveley Wood (Hunts.), which all lie within a few kilometers of one another. Thirty-seven traps were used across the seven sites (eight in Potton Wood, four in Buff Wood, and five in all others due to variation in wood size). Ecological profiles of these woods can be found in Catherall-Ostler (2022); further information on trap location is given in Supplementary Materials (Section A).

Burying beetles were caught using the method refined by Sun (2020): Japanese beetle traps were hung from the branch of a tree using a piece of string, suspended 1–2 m above the ground. Each trap consisted of a funnel, the top of which is covered by a baffle—two bisecting plastic dividers that increase beetle capture rate by deflecting beetles flying over the trap into the funnel (Fleming 1969). The funnel was screwed to a cylinder, and the cylinder was lined with small holes to allow the drainage of excess rainwater. Beetles were prevented from escaping due to the small size of the funnel opening, the steep slope of the funnel surface, and the baffle (Fleming et al. 1940). Each was filled to ¾ of its total volume with moistened MiracleGro All-Purpose Compost, and a freshly thawed mouse (c. 20–30 g) was placed on top of the soil. Beetles of all four species can breed successfully on mouse carcasses of the size used, i.e. all four species should be motivated to discover and attempt to breed on these carcasses. This mass range is also similar to the mass of the carrion base within these woods (Catherall-Ostler 2022).

All traps were placed in a similar microhabitat (hung off a tree branch ∼2 m high under a closed canopy). Traps were left for roughly two weeks before emptying and rebaiting, in common with other studies of British burying beetle populations (Easton 1979, Esh and Oxbrough 2021). A shorter timeframe would have resulted in fewer beetles per-trap, whilst a longer interval would have resulted in substantial within-trap deaths (Sun 2020). The mean length of time between trapping sessions was 14.8 d ([14.4, 15.2] 95% CI). Trapping began in late March in 2019 to ascertain when burying beetles emerged from overwintering: no beetles were recorded till the 18^th^ May trapping session. In 2020 the commencement of fieldwork was delayed due to COVID-19, with the first trapping session taking place on 23^rd^ July (and delayed further for some woods). Trapping in 2021 began in May and, in all three years ended in October when the number caught approached zero.

Each trap was fully emptied into a separate plastic box (17 × 12 × 6 cm) and brought back to the Department of Zoology, University of Cambridge. Each individual was then identified to the species level and anaesthetized with carbon dioxide, which allowed body size measurements. Pronotal width (and, in 2021, head width) was measured using Mitutoyo Digital Vernier calipers (resolution = 0.01 mm). Individuals were sexed using Easton’s (1979) observation that males possess an extra abdominal segment; sex ratio data is given in Supplementary Materials (Section B).

### Q1) Are Seasonal Patterns in Species Abundance Consistent with Seasonal Niche Partitioning?

As traps were left up for an average of two weeks (see above), the breeding season was divided up into fortnightly periods (e.g. early May, late May, early June, etc.). Due to COVID-19 related disruptions, not all time periods had the same number of trapping sessions per year (e.g. beetles were trapped from late May to early July onwards in 2019 and 2021, but in 2020 trapping only began in late July with further delays in Potton Wood). For this reason, simply plotting species counts across the seasons would not accurately represent seasonal patterns of variation in abundance. Hence, for each two week window, species counts were divided by the number of trapping events that had occurred in that window to produce a standardized measure of abundance that corrected for trapping effort. An assessment of whether proportional abundance ratios truly reflect interspecific encounter rate is given in Supplementary Materials (Section C).

### Q2) Do Coexisting Beetle Species Occupy Different Size Niches, as Measured by Pronotal and/or Head Width?

To test if species differed in pronotal width, an analysis of variance was performed on a linear model in which pronotal width was regressed against species identity. Tukey’s Honest Significant Difference procedure was then used to test for significant pairwise differences between the four species. The data were then split by species to test for sexual dimorphism and inter-annual consistency. For each species an analysis of variance was performed as above on a linear model in which pronotal width was regressed against sex and year of capture.

To evaluate which factors explained variation in head width, a linear model was constructed with a three-way interaction between pronotal width, sex, and species identity. The data were then split first by species and then by sex. Splitting by species allowed between-sex differences in head size allometries to be evaluated in each species; for each species an analysis of variance was performed on a linear model in which pronotal width was regressed against an interaction between pronotal width and sex. Splitting by sex allowed differences in the allometric scaling relationship between species to be identified (i.e. do males in species X have a steeper head size allometry than species Y?): for each sex, the estimated marginal means of each species’ allometry were compared using the *lsmeans* package (Lenth 2016).

### Q3) Does the Arrival of a Larger Species at a Trap Increase the Probability of Smaller Species Dying?

975 beetles were found dead in a trap but could still be identified to the species level. Binomial tests were used to test whether there was between-species variation in the likelihood of dying in the trap, with each species’ proportional abundance used to calculate the expected number of dead individuals that should have been found if there were no interspecific variation in the probability of death. To test whether *N. vespilloides* was more likely to die when heterospecifics were present in the trap, a logistic regression was performed via a generalized linear model (GLM) with a logit link and binomial distribution: considering only traps in which *N. vespilloides* was present, the presence/absence of a dead *N. vespilloides* was used as the response variable and regressed against the presence/absence of at least one heterospecific. We also tested whether the proportion of heterospecifics in a trap predicted the proportion of *N. vespilloides* found dead using a GLM constructed as above.

To evaluate which of the three species was responsible for causing excess *N. vespilloides* deaths, we also used a logistic regression GLM, as above, in which the presence/absence of *N. interruptus*, presence/absence of *N. investigator*, and presence/absence of *N. humator* were included as separate predictors of the risk of finding at least one dead *N. vespilloides*. Backwards stepwise elimination was then used to find the minimal model: likelihood ratio tests were then used to determine whether including a given predictor led to a significant decrease in deviance; if so, it was retained in the model; if not, it was removed from the model. This process continued until the minimal model was found.

If a significant relationship between a particular heterospecific and the risk of death was found, then it must be considered whether this is confounded by a relationship between the probability of finding that heterospecific in a trap and the total number of beetles in a trap. Perhaps heterospecifics are disproportionately likely to be found in high-density traps, and confinement with a large number of beetles increases the risk of death due to concomitant overheating or indirect competition for nutrients. Therefore we tested, using a logistic regression GLM, whether the proportional abundance of the heterospecific was predicted by the total number of beetles.

A two-sample *t*-test was performed to test if there was an overall difference in the mean pronotal width distribution of live and dead *N. vespilloides*. A binomial test was also used to determine whether there was between-sex variation in the likelihood of dying in the trap (*n* = number of deceased beetles; *x* = number of deceased males, expected chance of success = proportional sex ratio).

A further set of within-trap comparisons were also conducted, first for traps containing only *N. vespilloides* and then for traps containing *N. vespilloides* and heterospecifics. If deaths are caused by physical contests, then we would expect to find that dead *N. vespilloides* are smaller than living *N. vespilloides* in traps containing only other conspecifics. This is because in intraspecific contests smaller beetles are more likely to lose. For each dead *N. vespilloides*, the difference between its pronotal width and the mean pronotal width of all *N. vespilloides* in the trap was calculated. A one-sample t-test was used to test whether the mean of these differences differed from zero. This analysis was limited only to traps containing at least three *N. vespilloides*. If in traps also containing heterospecifics the deaths of *N. vespilloides* are caused by interspecific aggression, then we would not predict that dead *N. vespilloides* be smaller than the average *N. vespilloides* in that trap. The same analysis was therefore performed for traps also containing heterospecifics: for each dead *N. vespilloides*, the difference between its pronotal width and the mean pronotal width of all *N. vespilloides* in the trap was calculated. A one-sample *t-*test was used to test whether the mean of these differences differed from zero. This analysis was also limited to traps containing at least three *N. vespilloides*.

## Results

### Q1) Are Seasonal Patterns in Species Abundance Consistent with Seasonal Niche Partitioning?

**Fig. 1.**
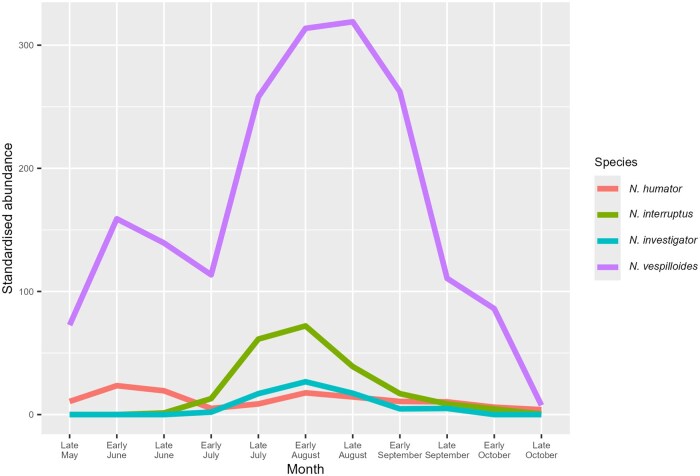
The abundance of the four burying beetle species across the three years of the project; “standardized abundance” refers to the correction for trapping effort described in the methods.

**Fig. 2.**
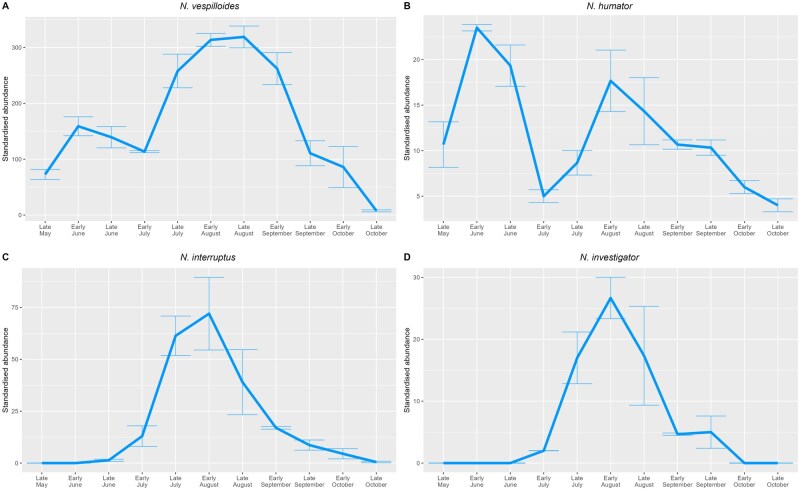
Abundance of the four burying beetle species across the three years of the project as per Fig. 1 but plotted separately for each species for enhanced clarity. “Standardized abundance” refers to the correction for trapping effort described in the methods. The standard deviation for each abundance estimate is represented by error bars.

There was no evidence for a strict seasonal separation between the *Nicrophorus* spp. inhabiting the seven woods studied, with all four burying beetle species active from July onwards (Fig. 1). Despite this, there was marked phenological variation between the species, with each exhibiting one of two distinct patterns in abundance. *N. vespilloides* and *N. humator* (Fig. 2A and B, respectively) both emerged in late May, peaked in June, and then again in August. *N. interruptus* and *N. investigator* (Fig. 2C and D, respectively), however, were absent before late June and then peaked once in August.

Comparing 2019 with 2021 allows the inter-annual consistency of these patterns to begin to be assessed. *N. vespilloides* peaked twice in both years and at reasonably similar times (first peak: late June [2019], early June [2021]; second peak: mid-August [2019], early September [2021]), as did *N. humator* (first peak: mid-June [both years]; second peak: August [both years]). *N. interruptus* and *N. investigator* both peaked once in August across all three years, but with a much lower abundance in 2019 than in the other two years. Dates of first capture are shown in Table 1; possible links with temperature are explored in Supplementary Materials (Section D). *N. vespilloides* was the most abundant species across the entire breeding season in all three years. The relative abundance of the other three species varied across the years (Table 2). When beetles were present in traps, carcasses were almost invariably buried.

**Table 1.** Dates of first capture across the two years in which trapping was conducted across the entire breeding season

Species	2019	2021
*N. vespilloides*	18^th^ May	27^th^ May
*N. humator*	18^th^ May	27^th^ May
*N. interruptus*	29^th^ June	19^th^ June
*N. investigator*	14^th^ July	15^th^ July

**Table 2.** The number of individuals of each species of burying beetle caught across the three years of the study

	*N. vespilloides*	*N. interruptus*	*N. humator*	*N. investigator*	Yearly total
2019	1,622	83	110	15	1,830
2020	1,336	335	102	103	1,876
2021	1,882	216	140	98	2,336
**Total**	**4,840**	**634**	**352**	**216**	**6,042**

### Q2) Do Coexisting Beetle Species Occupy Different Size Niches, as Measured by Pronotal and/or Head Width?

**Fig. 3.**
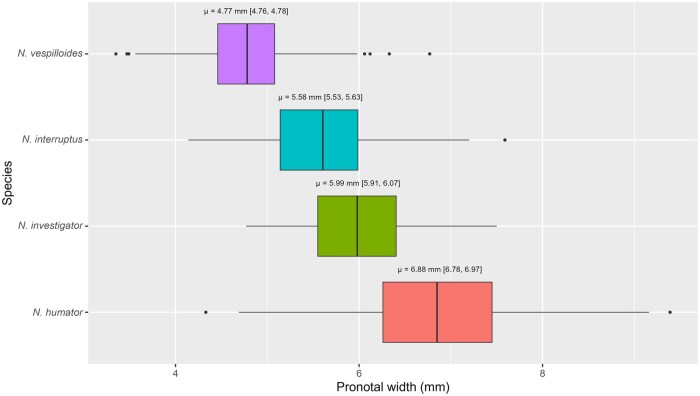
Pronotal widths of trapped *Nicrophorus* spp. For each species, the median is represented by the horizontal line and the first and third quartiles by the lower and upper hinges, respectively. The upper whisker terminates at the largest value, which is no further than 1.5 * IQR from the upper hinge, and the lower whisker terminates at the smallest value, which is at most 1.5 * IQR from the lower hinge. Each species mean (μ) and associated 95% confidence interval (in square brackets) is written above each box.

The species differed significantly in size as measured by the width of the pronotum (ANOVA: F_3, 5440_ = 2423, *P* < 0.001; Fig. 3), with all pairwise comparisons being significant (all *P* < 0.001, Tukey’s HSD), suggesting that each species occupies its own distinct size niche. *N. vespilloides*, *N. investigator*, and *N. humator* showed no inter-annual variation in pronotal width (ANOVA: F_2_=2.5, *P* = 0.08; F_2_=0.9, *P* = 0.40; and F_2_=0.15, *P* = 0.86, respectively), whilst there was significant inter-annual variation in *N. interruptus* (ANOVA: F_2_=4.2, *P* = 0.02) with individuals caught in 2019 being significantly larger than those caught in 2020 (Tukey multiple comparisons of means, *P* = 0.009).

There was no sexual size dimorphism in the pronotal widths of *N. vespilloides* or *N. humator* (ANOVA: F_1_=0.001, *P* = 0.97, and F_1_=0.007, *P* = 0.92, respectively), but in *N. interruptus* and *N. investigator* males were significantly larger than females (ANOVA: F_1_=20.6, *P* < 0.001, and F_1_=18.6, *P* < 0.001, respectively). The mean *N. interruptus* male was 5.69 mm [5.62, 5.77 95% CI], whereas the mean for females was 5.48 mm [5.42, 5.54]; in *N. investigator*, the mean male had a pronotal width of 6.14 mm [6.03, 6.25], whereas this was 5.81 [5.71, 5.91] mm in females.

**Fig. 4.**
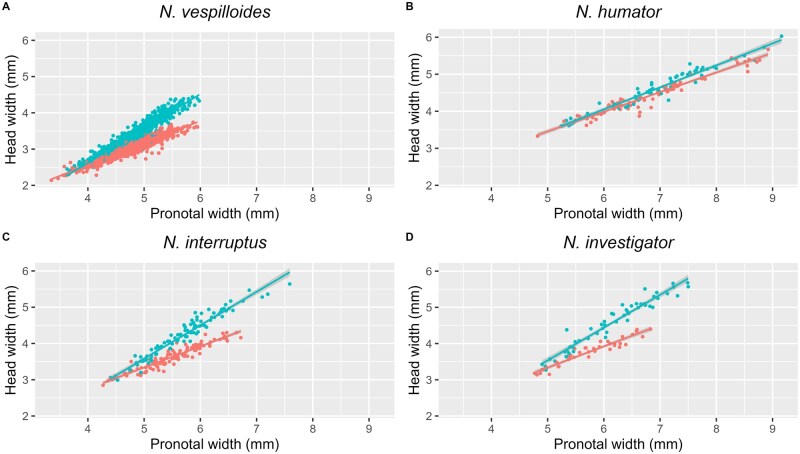
The relationship between pronotal width and head width plotted for each sex (blue = males, red = females) in the four burying beetle species (A–D). The 95% confidence interval is represented by grey shading around each regression line.

Variation in head width could be explained by a three-way interaction between sex, pronotal width, and species identity (ANOVA: F_9_ = 145, *P* < 0.001). When each species was considered separately, the slope of the head width—pronotal width allometry was consistently steeper in males than in females (Fig. 4; ANOVAs: *N. vespilloides: t*_1724_ = 29.3, *P* < 0.001; *N. interruptus: t*_202_ = 10.1, *P* < 0.001; *N. investigator: t*_86_ = 6.4, *P* < 0.001, and *N. humator: t*_136_ = 2.5, *P* = 0.01).

**Fig. 5.**
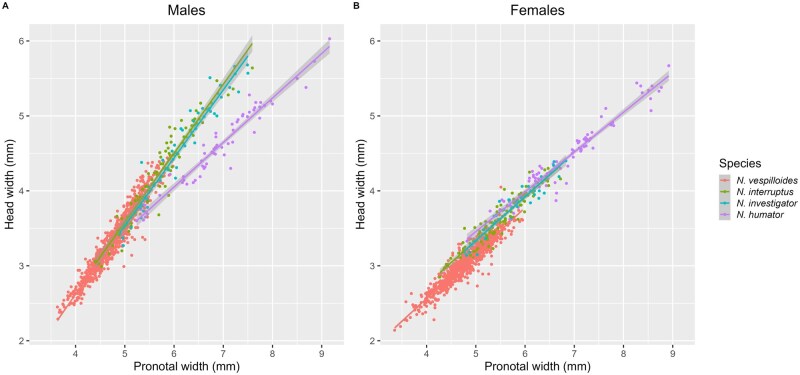
The relationship between pronotal width and head width for males of the four burying beetle species. The 95% confidence interval is represented by shading around each regression line.

Mean head widths differed significantly between all species (*N. vespilloides* < *N. interruptus* < *N. investigator* < *N. humator*; *P* < 0.001 for all pairwise comparisons). The slope of the head width—pronotal width allometry also differed between species: this relationship was significantly shallower in *N. humator* males than in any of the other species (Fig. 5), and in females was also shallower than the slope for *N. vespilloides* (Table 3).

**Table 3.** *t* and Tukey-adjusted *P*-values obtained from a comparison of the marginal means of each species’ linear trend line.

Males	*N. vespilloides*	*N. interruptus*	*N. investigator*	*N. humator*
*N. vespilloides*				
*N. interruptus*	*t * = 1.1, *P* = 0.70			
*N. investigator*	*t * = 1.6, *P* = 0.40	*t * = 0.5, *P* = 0.96		
*N. humator*	*t * = 16.2, *P* < 0.001	*t * = 11.2, *P* < 0.001	*t * = 9.8, *P* < 0.001	

Females				
				
*N. interruptus*	*t * = 1.2, *P* = 0.6			
*N. investigator*	*t * = 0.70, *P* = 0.90	*t* = ‐0.1, *P* = 1.0		
*N. humator*	*t *= 5.3, *P* < 0.0001	*t * = 2.5, *P* = 0.06	*t * = 1.9, *P* = 0.2	

### 3. Does the Arrival of a Larger Species at a Trap Increase the Probability of Smaller Species Dying?

975 of the beetles found were dead but could still be identified to species-level: dead beetles were disproportionately likely to be *N. vespilloides* (88% of corpses cf. 80% of all beetles caught; binomial test, *P* < 0.001). *N. humator*, *N. interruptus*, and *N. investigator* were all less likely to be found dead than the chance expectation (binomial tests, respectively: *P* < 0.001, *P* < 0.001, *P* = 0.02).

The probability of finding at least one dead *N. vespilloides* increased significantly when heterospecifics were present (23.6% chance without heterospecifics vs. 35.8% chance with; GLM: *z* = 3.5, *P* < 0.001). This effect appeared to be driven by the presence of *N. interruptus* (24.5% chance without vs. 40.1% chance with; GLM: *z* = 3.9, *P* < 0.001) rather than *N. humator* or *N. investigator* (likelihood ratio tests during minimal model selection, respectively: χ^2^_1_ = 0.33, *P* = 0.56; χ^2^_1_ = 0.78, *P* = 0.38). There was no evidence of a confounding relationship between the occurrence of *N. interruptus* and the total number of beetles in the trap (GLM: *z* = 1.3, *P* = 0.20). The proportion of *N. vespilloides* found dead also correlated with the proportion of heterospecifics in each trap (GLM: *z* = 6.2, *P*≪0.0001). Therefore, whilst the majority (52%) of the traps in which at least one *N. vespilloides* died contained only other *N. vespilloides*, the majority of deaths (54%) occurred in traps containing at least one heterospecific.

Of those that could be sexed, males were just as likely to be found dead as females (binomial test: *P* = 0.67). There was no difference in the overall mean pronotal width of dead vs. live *N. vespilloides* (4.78 mm [4.73, 4.82], n = 326 vs. 4.77 mm [4.76, 4.78], *n* = 4295; *t*-test: *t*_381_ = 0.28, *P* = 0.78). However, *N. vespilloides* that died in traps only containing other *N. vespilloides* had a significantly smaller pronotal width than the average *N. vespilloides* within that trap (average difference = 0.14 mm; one-sample *t*-test: *t*_106_ = −2.6, *P* = 0.005). In traps that contained *N. vespilloides* and also at least one heterospecific, there was no significant difference between the pronotal widths of dead *N. vespilloides* and the trap average for *N. vespilloides* (one-sample *t*-test: *t*_137_ = −1.13, *P* = 0.13).

## Discussion

For burying beetles living in the ancient woods of Bedfordshire, Cambridgeshire, and Huntingdonshire, interspecific coexistence might seem unlikely: our trapping data shows that all four species are attracted to the same type of carrion and will attempt to breed on it if the opportunity arises.

When species compete over similar resources, divergence in activity periods offers one potential mechanism to enable coexistence. Springett (1967) found a complete separation between the activity periods of *N. investigator* and *N. humator*, which he interpreted as a form of temporal niche partitioning. In our study area, the two species not only overlap in their periods of activity (as in Easton 1979), but also peak twice at the same times and show near-identical patterns of abundance from early July to early September. This is in sharp contrast to Esh and Oxbrough’s (2021) national study in which *N. vespilloides* remained consistently high throughout the growing season and the second peak for *N. humator* was in September. This suggests that the way the carrion niche is partitioned temporally varies qualitatively between burying beetle populations in the United Kingdom.

Whilst seasonal patterns of abundance in *N. vespilloides* and *N. humator* mirrored one another, *N. investigator* and *N. interruptus* followed a different pattern. They are completely absent until after the first peak of *N. vespilloides*/*N. humator*, only emerging from their overwintering chamber (Smith 2002) from late June onwards. Whilst this could be interpreted as *N. interruptus* and *N. investigator* avoiding competition from *N. humator* and *N. vespilloides*, the former peak at the same time as the latter’s second peak. Hence, in the burying beetle communities of the seven ancient woods studied here, there is little evidence for seasonal niche partitioning, unlike in other areas of the UK. Future work could investigate potential drivers of such differences in activity periods, such as avoiding high periods of fly activity (Scott 1998) or seasonal variation in carrion abundance (discussed in Issar 2021). We note that our finding of temporal coexistence at the seasonal level does not preclude the existence of more fine-grained temporal niche partitioning. Smaller species may have their activity periods restricted to certain parts of the day, as has been suggested for *N. guttula* (Cook et al. 2019) and *N. defodiens* (Trumbo and Bloch 2002); the potential for diel niche partitioning is a neglected topic in burying beetle ecological research and highly deserving of further investigation.

Our data support the hypothesis that the carrion niche is partitioned by size. Each species has a distinct size distribution, whether measured by pronotal width or head width. The pronotal width distributions reported for each species by Sun (2020) were both replicated and made more precise by more than doubling the sample size. Whilst in all four species head width scaled positively with body size, this relationship was significantly shallower for *N. humator*: perhaps due to its large size, *N. humator* would not need the “disproportionately” large jaws that the other three smaller species are selected to have.

If head width is a better proxy of competitive ability than pronotal width, then we would expect sex differences in its allometric slope due to males experiencing more intense selection for fighting ability than females (Eggert and Müller 1997, Trumbo 2006). We discovered a sexual dimorphism in head width in *N. humator*, *N. interruptus*, and *N. investigator* that is similar to the relationship recently discovered in *N. vespilloides* (Smith et al. 2024). The distant phylogenetic relationships between these species (Sikes and Venables 2013) imply that this is an ancient and widely shared feature of *Nicrophorus*. If this is correct, it provides an explanation for the general lack of body size dimorphism in the genus despite males experiencing stronger selection on fighting ability (Eggert and Müller 1997, Trumbo 2006).

We also found, however, that in our sample male *N. interruptus* and *N. investigator* were significantly larger than females, as measured by pronotal width. This was unexpected, as sexual dimorphism in body size is considered uniformly absent from burying beetles (Sikes et al. 2002, Gibbs et al. 2008, Al Shareefi and Cotter 2019), presumably as both sexes have to carry out similar tasks. Only one, seemingly anomalous, prior report of sexual size dimorphism was located (Otronen 1988), in which *N. vespilloides* and *N. investigator* females were both shown to be longer (as measured by elytral length) than males; male-biased sexual size dimorphism is rare in insects generally (Teder and Tammaru 2005). Whilst it is tempting to ascribe this dimorphism to increased selection on fighting ability in males (Andersson 1994), why this would only affect these two intermediately-sized species in particular is unclear.

The large numbers of dead *N. vespilloides* found that showed signs of physical damage, in both traps containing only *N. vespilloides* and those containing a mix of *Nicrophorus* spp., highlights the intensity of intraspecific and interspecific competition that burying beetles experience. Whilst the confinement of beetles within traps may have artificially inflated the death rate (as in nature weaker beetles would have the option to leave a carcass in “retreat”), deaths were not distributed randomly between traps. Consistent with the idea that larger species are competitively superior to smaller species, the smallest species (*N. vespilloides*) was disproportionately likely to be found dead in traps. Whilst causes other than susceptibility to interspecific competition are possible (e.g. larger insect species do often live longer (Holm et al. 2016); smaller species may be more affected by the negative physiological consequences of overcrowding), the high frequency of deantennated, amputated, and decapitated beetles suggests these deaths represent the consequences of poor competitive ability at highly contested carcasses. The presence of larger species also significantly increased the probability of an *N. vespilloides* being found dead. Fighting as a frequent cause of death also explains the lack of size differences between surviving and deceased *N. vespilloides* in traps where heterospecifics are present, as the majority of individual heterospecifics are significantly larger than the majority of individual *N. vespilloides*. However, in traps containing only *N. vespilloides*, smaller individuals now are more likely to die, as the sizes of combatants in intraspecific fights are more similar.

The species that had the strongest association with dead *N. vespilloides* is the species closest in size—*N. interruptus*. The presence of *N. interruptus* led to a 64% increase in the risk of at least one *N. vespilloides* being dead, compared to traps without *N. interruptus*. This provides further evidence for the size-based niche partitioning hypothesis in burying beetles: as *N. vespilloides* is most similar in size to *N. interruptus*, this hypothesis predicts it experiences a higher level of niche overlap with *N. interruptus* than with other heterospecifics. This in turn makes them more likely to enter into physical conflict with each other.

This negative association between *N. vespilloides* and *N. interruptus* in our study has not been reported before, but this is unsurprising given the national rarity of *N. interruptus*. Classed as possibly declining and nationally scarce (Hyman and Parsons 1994, Lane 2020), *N. interruptus* is also rare in Europe (Dekeirsschieter et al. 2011), including a near-total absence from the island of Ireland (O’Hanlon et al. 2020): unexpectedly, *N. interruptus* was the second-most abundant in our study area. Esh and Oxbrough’s (2021) trapping of carrion beetles at 24 sites across the UK caught only six *N. interruptus*, in comparison to 4750 *N. vespilloides*, 192 *N. investigator*, and 128 *N. humator*.

*N. investigator* is morphologically and phenologically similar to *N. interruptus* but, in contrast, is abundant in the UK and Europe (Dekeirsschieter et al. 2011, Lane 2020, O’Hanlon et al. 2020) whilst being comparatively rare in our study area. The rarity of *N. interruptus* nationally is unlikely to be an artifact caused by misidentification: there is a pronounced difference between the elytral markings of *N. interruptus* and *N. investigator*, more so than between many others in the genus. Targeted trapping could reveal the size of this *N. interruptus* hotspot and help identify the cause: one potential explanation is that an as-yet unidentified ecological variable that nationally favors *N. investigator* at the expense of *N. interruptus* differs in these woods. Understanding why some insects are rare whilst similar species are common is, however, notoriously difficult (Howard and Hall 2019), with even seemingly trivial differences in habitat sometimes enough to cause extinction (e.g. Thomas et al., 2001).

Climate change is causing worrying phenological disruption across taxa (Badeck et al. 2004), with general global trends complicated by between-species and between-population variation (Parmesan 2007, Gutiérrez and Wilson 2021). Comprehensively understanding these changes is essential for identifying at-risk taxa (Roy et al. 2015) but requires recording and publishing phenological data as a matter of routine (Ovaskainen et al. 2020). The publication of such data is particularly important for carrion beetles given their important role in forensic entomology (Michaud et al. 2010).

Whilst we believe this trapping program has yielded useful data on the broad-scale demographics of British burying beetle populations, more fine-grained future work could improve the accuracy of the magnitude of our abundance estimates. We kept the trapping window and trapping density constant throughout the year to ensure consistency in sampling effort. This may have caused us to artificially underestimate the abundance of *Nicrophorus* in the summer months, when carcasses decay quicker: a carcass left out for 14 days in the spring/autumn will attract burying beetles for a longer proportion of the trapping window than a carcass in the summer. The increased abundance seen for all species in the summer will also likely reduce the average elapsed time till the carcass is first discovered by a *Nicrophorus*. After discovering a carcass, burying beetles attempt to mask the odorants that could attract competitors (Trumbo et al. 2021). Quicker discovery in summer months could also therefore lead to an underestimation of abundance: whilst this will not affect the timings of abundance peaks observed here, it will affect their magnitude. Finally, the use of only a limited mass range of mouse carcasses could lead to an underestimation of abundance for any species that preferentially search for smaller or larger carcasses, or those of a different species. A separate limitation of this program was its use of a single habitat type: whilst in Britain *N. vespilloides* has a significant preference for woodland habitats, *N. interruptus* and *N. humator* are also frequently found in unforested open habitats (Esh and Oxbrough 2021). Whether different habitat preferences result from a selective pressure for competition avoidance or are instead due to differing tolerances for abiotic factors (e.g. soil compaction, temperature) remains unclear (Smith and Heese 1995, Scott 1998, Kocárek 2001, Esh and Oxbrough 2021).

Nevertheless, comparisons between the dates of activity recorded here and those in other studies reveal major between-population variation in burying beetles. For example, Springett (1967) records *N. humator* in Northumberland (UK) as becoming active in late April, peaking in May before declining to a four/five week total absence (early August to mid-September). A second peak then occurred in early October. This is a markedly different phenology to that observed here, where *N. humator* emerged in May, peaked in early June, and then again in August without any period of total absence. Dekeirsschieter et al’s (2011) European review records both *N. interruptus* and *N. investigator* becoming active in April (c.f. late June in our study area), whilst Easton (1979) records emergence dates in Stirlingshire (UK) for *N. vespilloides* that are consistently earlier by a month than our study area . Whilst not previously commented upon, the magnitude of between-population variation in phenology appears to be remarkable in burying beetles: further work could test whether this variation is due to plastic responses to climatic variation or evolutionary divergence in life history strategy. Climate change is also affecting voltinism (Tobin et al. 2008); the number of annual peaks in abundance are often interpreted as indicating voltinism in burying beetles (Scott 1998). Interestingly, whilst we found one peak in *N. investigator*, Springett (1967) recorded two; Royle and Hopwood's (2017) Cornish (UK) population of *N. vespilloides* appears to peak three times.

## Supplementary Material

nvaf087_Supplementary_Data

## Data Availability

All data associated with this publication are available at https://doi.org/10.17863/CAM.121611.
