# Supplementary material for: Size-based niche partitioning permits coexistence in natural populations of Nicrophorus spp
Source: Environ Entomol. 2025 Oct 28;54(6):1412–22. doi: 10.1093/ee/nvaf087 (PMC12716276; doi:10.1093/ee/nvaf087)
Supplement: nvaf087_Supplementary_Data [file nvaf087_supplementary_data.zip › Alt text.docx]

**Figure 1**

Alt text: A line graph displaying standardised abundance (y-axis) across the year (fortnightly blocks beginning in Early May and ending in Late October, x-axis). Each of the four species’ abundance is plotted in a different colour.

**Figure 2**

Alt text: A panel figure showing four separate line graphs for each of the fours species. For each species, standardised abundance (y-axis) is plotted against the time of year (fortnightly blocks beginning in Early May and ending in Late October, x-axis). Standard deviations for each fortnightly estimate are represented by error bars.

**Figure 3**

Alt text: A box-and-whisker plot showing how the pronotal width distribution differs between the four species of *Nicrophorus* studied. Each row corresponds to one of the four species; for each species, the median, first and third quartiles, largest value which is no further than 1.5 * IQR from the upper hinge and the smallest value which is at most 1.5 * IQR from the lower hinge are shown. Each species mean and associated 95% confidence interval is also written above each box.

**Figure 4**
Alt text: A panel figure showing four separate scattergraphs for each of the four species. For each species, head width (y-axis) is plotted against pronotal width (x-axis), with male and female individuals coloured differently. Regression lines are plotted for males and females on each graph.

**Figure 5**

Alt text: A scattergraph showing the relationship between head width (y-axis) and pronotal width (x-axis) for the four species studied here; all four are shown on the same graph but coloured differently. Regression lines are plotted for each of the four species.

**Figure S1**

Alt text: A satellite image (from Google Earth) that shows the seven woodlands at which trapping occurred in this study. The trap locations are shown by a yellow circle. A scale bar shows that the woods are close together (i.e. most are few km from each other).

**Figure S2**

Alt text: A line graph showing weekly average maximum temperatures for Cambridge NIAB weather station at the times of year when burying beetles are active (i.e. March – October). Each year is plotted in a different colour (2019, 2020, and 2021).

**Figure S3**

Alt text: A line graph showing how the proportional sex ratio varies across the year (averaged over the three years of the study). A red dashed line runs horizontally across the plot at 0.5, showing the null expectation (50:50 sex ratio).
