# Supplementary material for: Size-based niche partitioning permits coexistence in natural populations of Nicrophorus spp
Source: Environ Entomol. 2025 Oct 28;54(6):1412–22. doi: 10.1093/ee/nvaf087 (PMC12716276; doi:10.1093/ee/nvaf087)
Supplement: nvaf087_Supplementary_Data [file nvaf087_supplementary_data.zip › Supplementary materials.docx]

**Supplementary materials**

1. **Trapping strategy**

The number of traps set per woodland was scaled according to the area of each wood (Woodcock, 2005) with one trap for every 10 ha of woodland. This led to the placement of eight traps in Potton and five in Cockayne Hatley, Gamlingay, Hayley, Waresley and Weaveley. According to this scaling rule, two traps should have been set in Buff Wood but, to avoid setting insufficiently few traps to sample the community accurately (Leather & Watt, 2008; Obrtel, 1971), four traps were set instead. There was no evidence that trap number per woodland was correlated with either *Nicrophorus* species diversity or abundance.

Practical constraints (i.e. proximity to paths, avoiding areas of tree felling) prevented random trap placement, but we aimed to spread traps around the woods in a single ‘circumference’ to ensure representative sampling and between-trap independence (Digweed, 1995). The same exact trapping sites were used for each year of the study (Figure S1). An analysis of between-wood differences in burying beetle community structure and differences in phenotype distributions can be found in Catherall-Ostler (forthcoming).

A sceptic may be concerned that an intensive programme of removal sampling for three years may affect population dynamics and cause a reduction in population size. We have three reasons to think this is not the case. Firstly, previous work in our lab suggests that we catch approximately only 10% of the population each year (Sun, 2020). Secondly, trapping has been continuing for seven years at some of these woods with no evidence of a decline in population size. Thirdly, the consensus in the literature is that far more burying beetles are born each year than can successfully breed on a carcass (Scott, 1998) – so removing ~ 10% each year is not going to reduce overall population size.

**B) Sex ratios**
When looking at adult *Nicrophorus* sex ratios there is marked variation between species and populations, with trapping studies reporting significant male biases (Easton, 1979; Springett, 1967), significant female biases (Sikes, 1996; Terrón et al., 1991; Trumbo, 1990; D. S. Wilson & Knollenberg, 1984) as well as ratios at equality. The extrinsic and intrinsic factors that drive this variation in sampled sex ratios is poorly understood (Sikes, 1996). An additional complication is that trap-derived burying beetle sex ratios are known to vary across the breeding season, and these patterns likewise vary between studies (Easton, 1979; Royle & Hopwood, 2017; Springett, 1967). By virtue of its three-year duration, this study allows a better estimation of each species’ sex ratio as well as its inter-annual consistency.

As in Supplementary Materials Section C, there are also reasons to think that the encounter rate between the sexes at a carcass may not be a simple function of the population sex ratio. Firstly, upon arrival at a carcass males invest heavily in mate-attractant pheromone emission, a behaviour that is entirely absent in females (Haberer et al., 2011). If these pheromones are highly effective at attracting females, traps containing a small number of beetles should have a female-biased sex ratio and vice-versa. Secondly, whilst females can only gain reproductive success by laying eggs at a carcass, a male can gain paternity anywhere through non-carcass-based signalling (Eggert and Müller 1989a, 1989b; Müller and Eggert 1987). Hence we might expect that females would invest more in searching for a carcass than males, but the only comparison of first-arriving beetles located found no such bias (Hopwood et al. 2016), albeit with a modest sample size. We used our much larger sample to test whether sex ratios differed across different levels of trap occupancy.

*Methods:*The proportional sex ratio for each species was calculated by dividing the total number of males caught by the total number of males and females caught (K. Wilson & Hardy, 2002). Binomial tests were performed to test if this differed from the expected ratio of 0.5. To test for inter-annual variation in the sex ratio, a logistic regression was performed for each species: likelihood ratio tests were used to assess if including year led to a significant decrease in deviance.

To test whether any sex ratio bias observed in the field was due to a true evolutionary departure from the Fisher condition, or whether it was due to environmental ‘filters’ that altered the number of males and females arriving at a trap (e.g. more males dying in contests, or females being more motivated to find a trap), the sex ratio of laboratory populations founded by field beetles from the seven woods studied here was calculated. Details are given of how the populations were created and maintained in Catherall-Ostler (2022). All individuals in the second generation surviving to eclosion were sexed – a total of 4358 beetles from 225 families. A binomial test was used to see if the overall population sex ratio differed from the 0.5 expectation.

I also tested whether the family sex ratio varies according to maternal or paternal size: sex allocation theory predicts that, when mating with a high quality male, females may be selected to produce male-biased broods (Charnov, 1982). Following Wilson and Hardy's (2002) procedure for the analysis of family sex ratio data, I used a logistic regression in which each families’ sex ratio was weighted by family size. A maximal model was constructed in which maternal and paternal pronotal width interacted, with variation in carcass size controlled for. Backwards stepwise elimination was then used to find the minimal model, with likelihood ratio tests used to assess whether any of these covariates were significant.

To test if the between-sex encounter rate differed from the chance expectation, I tested whether the sex ratio differed across different levels of trap occupancy. The same procedure was followed as for the question above addressing differences in the between-species encounter rate – i.e. binomial tests were performed (*n* = number of males and females, *x* = number of males, expected chance of success = proportional sex ratio) for, successively, traps containing one, two, three and four burying beetles. For each level of trap occupancy, one test was performed in which all four species were pooled together and another which considered *N. vespilloides* separately.

*Results:*

The proportional sex ratio of trapped *N. vespilloides* and *N. humator* was 0.470 ([0.454, 0.485] 95% CI) and 0.431 ([0.377, 0.486] respectively; in both species this female bias was small but statistically significant (binomial tests, respectively: p<0.0001, n=4263; p=0.01, n=334). The sex ratios of *N. interruptus* (0.470 [0.429, 0.511]) and N. investigator (0.551 [0.481, 0.621]) were not significantly different from equality (binomial tests, respectively: p=0.16, n=585; p=0.17, n=207). In none of the four species was there any inter-annual variation in the sex ratio (likelihood ratio tests, *N. vespilloides*: χ²_2_=1.4, p=0.5; *N. humator*: χ²_2_=0.46, p=0.80; N. interruptus: χ²_2_=2.0, p=0.37; N. investigator: χ²_2_=0.07, p=0.96).

The female-bias observed in field-caught *N. vespilloides* persisted in the laboratory populations: the proportional sex ratio was 0.481 ([0.466, 0.496]; 2262 females and 2096 males). No variation in family sex ratios was explained by paternal size, maternal size, or their interaction (LRTs, respectively: χ_1_²= 0.18, p= 0.67; χ_1_²= 0.07, p= 0.79; χ²_1_= 0.03, p= 0.85).

The sex ratio of *N. vespilloides* varied across the breeding season; as shown in Figure S2, there was a slight male bias until late June. Between late June and early July the ratio sharply dipped until late August before rising to equality in early September.

The presence of males and females across different levels of trap occupancy was consistent with the chance expectation, with the exception of a female bias in single-occupancy traps. One possibility is that this bias is due to females being more likely to find the carcass before males (contra Hopwood et al., 2016a), with their dependence on carcasses for reproduction selecting for increased carcass-discovery effort. As mites depend on carcasses for reproduction, this would suggest their apparent preference for female beetles observed here may be adaptive. Alternatively, the female bias in single-occupancy traps may be simply due to the fact that females do not engage in the mate-attractant signalling that males do.

**C) Interspecific encounter rate**A common assumption in studies of burying beetle community ecology is that the interspecific encounter rate at carcasses is a simple function of species’ proportional abundance (e.g. (Koulianos & Schwarz, 2000; Trumbo & Thomas, 1998). For example, if 80% of individuals in a two-species burying beetle community are *N. vespilloides* and the remaining 20% are *N. humator*, the probability that the first (or subsequent) beetle to arrive at a carcass is *N. vespilloides* is assumed to be 0.8. At least two aspects of burying beetle biology could make this assumption questionable. Firstly, the order of species arrival may be non-random, with some species quicker to arrive than others. As larger insects are often better fliers (Kaufmann et al., 2013), it might be suggested that the largest species, *N. humator*, may be disproportionately likely to arrive first at carcasses (but Attisano & Kilner, (2015) found no relationship between body size and flight performance). Alternatively, smaller species may invest more in prompt carcass discovery as a competition-avoidance mechanism (Trumbo 1991; Woodard 2006)

Secondly, between-species differences in either pheromone emission or detection could lead to the identity of the species currently present on the carcass biasing the distribution of subsequent arrivals. If the pheromone output of smaller species is low (Byers, 2005; Pureswaran & Borden, 2003), carcasses they discover may be less likely to see further arrivals compared to those discovered by larger species. Alternatively, asymmetries in pheromonal eavesdropping between small and large species may lead to the opposite pattern: *N. humator* is attracted to the pheromones *N. vespilloides* emits upon carcass discovery, but *N. vespilloides* is not attracted to *N. humator* (Haberer et al., 2011). Competitively dominant larger species could hence invest less time and energy into initial carcass discovery and instead ‘free-ride’ off the discoveries made by smaller species, creating producer-scrounger dynamics (Trumbo and Bloch 2002). If either or both of these processes generate non-randomness in the species encounter rate, this could change the average competitive environment faced by each species. Hence we tested if the distribution of species across traps of different occupancies differed from the chance expectation.

*Methods:*
Considering the 129 traps that contained a single beetle, the number of traps in which the lone beetle was an *N. vespilloides* was counted, and the procedure repeated for *N. humator*, *N. interruptus*, and *N. investigator*. Binomial tests were then used to see if these frequencies departed from the null expectation given the species’ proportional abundance. For example, *N. vespilloides* was the lone resident in 93 of the 129 single-beetle traps, and across all traps had a proportional abundance of 0.801: hence a binomial test was performed with *n* = 129 and *x* = 93 and a chance of success of 0.801. This procedure was then repeated for traps that contained two (*n* = 89), three (*n* = 84) and four (*n* = 60) burying beetles.

E.g. 89 traps contained two beetles (*n* = 89*2 = 178 beetles) and 140 of these were *N. vespilloides*. As *N. vespilloides* has an overall proportional abundance of 0.801, a binomial test was performed with *n* = 178 and *x* = 140 and a chance of success of 0.801. This was then repeated for traps with three beetles and traps with four beetles. Four was chosen as the termination point as below this sample sizes begin to become too small. To minimise the number of tests performed and ensure adequate sample sizes, the three larger species (*N. humator*, *N. interruptus*, and *N. investigator*) were counted together for traps containing two, three and four burying beetles.

*Results and discussion:*

When considering the traps where only one beetle had so far arrived at the carcass, the individual was disproportionately likely to be *N. humator* (binomial test: p<< 0.0001) and less likely than chance to be *N. vespilloides* (binomial test: p = 0.03), with the frequency of lone *N. interruptus* and *N. investigator* being no different to the null expectation (binomial tests, respectively: p = 0.15, p =0.1). Looking at traps where two, three and four beetles were present showed no evidence that abundance of *N. vespilloides* relative to the abundance of the three larger species differed from the chance expectation (binomial tests, respectively: n = 178 beetles, p = 0.64; n = 252, p = 0.27; n =240, p = 0.37).

With respect to species identity, the arrival of burying beetles at traps appears to be almost random. This provides empirical support for the common assumption that differences in species encounter rate between two populations can be inferred from comparing differences in proportional abundance (Koulianos & Schwarz, 2000; Trumbo & Thomas, 1998). The only non-randomness detected in arrival patterns was for single occupancy traps, in which *N. humator* and *N. vespilloides* were over represented and under-represented respectively. This fact is consistent with several alternate hypotheses: for example, the larger *N. humator* may possess superior olfactory or flight capacities and hence be the first to arrive at carcasses relative to the small *N. vespilloides*. This would be the reverse of the difference in resource location efficiency identified by Trumbo & Bloch (2002), who found that the smaller *N. defodiens* located carcasses quicker than the larger *N. orbicollis*. Alternatively the over-representation of *N. humator* in single-occupancy traps may be due to interspecific variation in signalling effort (i.e. *N. vespilloides* may invest more in pheremonal signalling than *N. humator* and hence be less likely to be found alone in traps; Chemnitz et al., 2015; Walling et al., 2009). Future work that addressed these and other possibilities would increase our understanding of the diverse ways in which these ecologically similar species co-exist despite intense interspecific competition.

**D) Temperature records**

Daily maximum air temperature, averaged by week, is shown for March – October across the three years of the study in Figure S3.

By eye, this seems to correlate with the variation in first emergence dates of different *Nicrophorus* species between 2019 and 2021 (Table 1). April - May 2019 was warmer than April – May 2021, and both *N. vespilloides* and *N. humator* emerged earlier in 2019 than in 2021. However early June 2021 was warmer than early June 2019, and *N. interruptus* emerged earlier in 2021 than 2019. *N. investigator* emerged in mid-July at the same point in 2019 and 2021, and the temperature in the weeks leading up to this point was relatively similar between the two years.

**Bibliography**

Attisano, A., & Kilner, R. M. (2015). Parental effects and flight behaviour in the burying beetle, Nicrophorus vespilloides. *Animal Behaviour*, *108*, 91–100.

Byers, J. A. (2005). A cost of alarm pheromone production in cotton aphids, Aphis gossypii. *Naturwissenschaften*, *92*(2), 69–72.

Catherall-Ostler, A. (2022). *Evolution in a fragmented world: Phenotypic and genetic divergence amongst neighbouring populations of the burying beetle Nicrophorus vespilloides* [PhD Thesis, University of Cambridge]. https://www.repository.cam.ac.uk/handle/1810/354300

Charnov, E. L. (1982). *The theory of sex allocation* (Vol. 18). Princeton university press. https://books.google.co.uk/books?hl=en&lr=&id=-uQ9DwAAQBAJ&oi=fnd&pg=PP9&dq=Charnov,+E.+L.+1982.+The+Theory+of+Sex+Allocation.+Princeton+University+Press.&ots=JyjtBCpgUo&sig=gUcDIm1mHNKfp9JN31WcAZ1Sf3w

Chemnitz, J., Jentschke, P. C., Ayasse, M., & Steiger, S. (2015). Beyond species recognition: Somatic state affects long-distance sex pheromone communication. *Proceedings of the Royal Society B: Biological Sciences*, *282*(1812), 20150832. https://doi.org/10.1098/rspb.2015.0832

Digweed, S. C. (1995). Digging out the" digging-in effect" of pitfall traps: Influences of de pletion and disturbance on catches of ground beetles (Coleoptera: Cara bidae). *Pedobiologia*, *39*, 561–576. Google Scholar. https://doi.org/10.1016/s0031-4056(24)00225-7

Easton, C. (1979). *The ecology of burying beetles (Necrophorus: Coleoptera, Silphidae)* [PhD Thesis, University of Glasgow]. http://encore.lib.gla.ac.uk/iii/encore/record/C__Rb1628976

Eggert, & Müller. (1989a). Mating success of pheromone-emitting Necrophorus males: Do attracted females discriminate against resource owners? *Behaviour*, 248–257.

Eggert, & Müller. (1989b). Pheromone-mediated attraction in burying beetles. *Ecological Entomology*, *14*(2), 235–237.

Haberer, W., Schmitt, T., Schreier, P., & Müller, J. K. (2011). Intended and unintended receivers of the male pheromones of the burying beetles Nicrophorus humator and Nicrophorus vespilloides. *Entomologia Experimentalis et Applicata*, *140*(2), 122–126. https://doi.org/10.1111/j.1570-7458.2011.01143.x

Hopwood, P. E., Moore, A. J., Tregenza, T., & Royle, N. J. (2016a). Niche variation and the maintenance of variation in body size in a burying beetle. *Ecological Entomology*, *41*(1), 96–104. https://doi.org/10.1111/een.12275

Hopwood, P. E., Moore, A. J., Tregenza, T., & Royle, N. J. (2016b). The effect of size and sex ratio experiences on reproductive competition in Nicrophorus vespilloides burying beetles in the wild. *Journal of Evolutionary Biology*, *29*(3), 541–550. https://doi.org/10.1111/jeb.12803

Kaufmann, C., Reim, C., & Blanckenhorn, W. U. (2013). Size-dependent insect flight energetics at different sugar supplies. *Biological Journal of the Linnean Society*, *108*(3), 565–578. https://doi.org/10.1111/j.1095-8312.2012.02042.x

Koulianos, S., & Schwarz, H. H. (2000). Probability of Intra- and Interspecific Encounters, and the Duration of Parental Care in Nicrophorus investigator (Coleoptera: Silphidae). *Annals of the Entomological Society of America*, *93*(4), 836–840. https://doi.org/10.1603/0013-8746(2000)093[0836:POIAIE]2.0.CO;2

Leather, S. R., & Watt, A. D. (2008). Sampling theory and practice. In S. R. Leather (Ed.), *Insect Sampling in Forest Ecosystems*. John Wiley & Sons; Google Books.

Müller, J. K., & Eggert, A. K. (1987). Effects of carrion-independent pheromone emission by male burying beetles (Silphidae: Necrophorus). *Ethology*, *76*(4), 297–304.

Obrtel, R. (1971). Number of pitfall traps in relation to the structure of the catch of soil surface Coleoptera. *Acta Entomologica Bohemoslovaca*, *68*(5), 300–309. agris.fao.org.

Pureswaran, D. S., & Borden, J. H. (2003). Is bigger better? Size and pheromone production in the mountain pine beetle, Dendroctonus ponderosae Hopkins (Coleoptera: Scolytidae). *Journal of Insect Behavior*, *16*(6), 765–782.

Royle, N. J., & Hopwood, P. E. (2017). Covetable Corpses and Plastic Beetles—The Socioecological Behavior of Burying Beetles. In M. Naguib, J. Podos, L. W. Simmons, L. Barrett, S. D. Healy, & M. Zuk (Eds.), *Advances in the Study of Behavior* (Vol. 49, pp. 101–146). Academic Press. http://www.sciencedirect.com/science/article/pii/S0065345416300201

Scott, M. P. (1998). The Ecology and Behavior of Burying Beetles. *Annual Review of Entomology*, *43*(1), 595–618. https://doi.org/10.1146/annurev.ento.43.1.595

Sikes, D. S. (1996). The natural history of Nicrophorus nigrita, a western Nearctic species (Coleoptera: Silphidae). *Pan Pacific Entomologist*, *72*(2), 70–81.

Springett, B. P. (1967). *The biology of Necrophorus (Col.) and the mortality of terns (Sterna); an ecological study* [PhD Thesis, Durham University]. https://etheses.dur.ac.uk/9084/

Sun, S.-J. (2020). *On the ecological transitions between parasitism and mutualism* [PhD Thesis, University of Cambridge]. https://www.repository.cam.ac.uk/handle/1810/298740

Terrón, R. A., Anduaga, S., & Morón, M. A. (1991). Análisis de la coleopterofauna necrófila de la Reserva de la Biosfera «La Michilia», Durango, México. *Folia Entomológica Mexicana*, *81*, 315–324.

Trumbo, S. T. (1990). Reproductive Success, Phenology and Biogeography of Burying Beetles (Silphidae, Nicrophorus). *The American Midland Naturalist*, *124*(1), 1–11. https://doi.org/10.2307/2426074

Trumbo, S. T. (1991). Reproductive Benefits and the Duration of Paternal Care in a Biparental Burying Beetle, Necrophorus Orbicollis. *Behaviour*, *117*(1–2), 82–105. https://doi.org/10.1163/156853991X00139

Trumbo, S. T., & Bloch, P. L. (2002). Competition between Nicrophorus orbicollis and N. defodiens: Resource locating efficiency and temporal partitioning. *Northeastern Naturalist*, 13–26.

Trumbo, S. T., & Thomas, S. (1998). Burying beetles (Coleoptera: Silphidae) of the Apostle Islands, Wisconsin: species diversity, population density and body size. *The Great Lakes Entomologist*, *31*(2), 1.

Trumbo, S., & Thomas, S. (1998). Burying Beetles (Coleoptera: Silphidae) of the Apostle Islands, Wisconsin: Species Diversity, Population Density and Body Size. *The Great Lakes Entomologist*, *31*(2). https://scholar.valpo.edu/tgle/vol31/iss2/1

Walling, C. A., Stamper, C. E., Salisbury, C. L., & Moore, A. J. (2009). Experience does not alter alternative mating tactics in the burying beetle Nicrophorus vespilloides. *Behavioral Ecology*, *20*(1), 153–159.

Wilson, D. S., & Knollenberg, W. G. (1984). Food discrimination and ovarian development in burying beetles (Coleoptera: Silphidae: Nicrophorus). *Annals of the Entomological Society of America*, *77*(2), 165–170.

Wilson, K., & Hardy, I. C. (2002). Statistical analysis of sex ratios: An introduction. *Sex Ratios: Concepts and Research Methods*, *1*, 48–92.

Woodard, C. (2006). *Odor masking of a vertebrate carcass by a burying beetle (Nicrophorus marginatus)* [PhD Thesis]. Texas Tech University.

Woodcock, B. (2005). Pitfall trapping in ecological studies. In S. R. Leather (Ed.), *Insect Sampling in Forest Ecosystems* (1st ed.). Wiley. https://doi.org/10.1002/9780470750513
